# Galectin-9/Tim-3 pathway mediates dopaminergic neurodegeneration in MPTP-induced mouse model of Parkinson’s disease

**DOI:** 10.3389/fnmol.2022.1046992

**Published:** 2022-11-21

**Authors:** Qinyu Peng, Guoxin Zhang, Xiaodi Guo, Lijun Dai, Min Xiong, Zhaohui Zhang, Liam Chen, Zhentao Zhang

**Affiliations:** ^1^Department of Neurology, Renmin Hospital of Wuhan University, Wuhan, China; ^2^Department of Laboratory Medicine and Pathology, University of Minnesota Medical School, Minneapolis, MN, United States

**Keywords:** neurodegenerative diseases, Galectin-9, neuroinflammation, mitochondrial dysfunction, Parkinson’s disease

## Abstract

Galectin-9 (Gal-9) is a crucial immunoregulatory mediator in the central nervous system. Microglial activation and neuroinflammation play a key role in the degeneration of dopaminergic neurons in the substantia nigra (SN) in Parkinson’s disease (PD). However, it remains unknown whether Gal-9 is involved in the pathogenesis of PD. We found that MPP^+^ treatment promoted the expression of Gal-9 and pro-inflammatory cytokines (IL-6, IL-1β, TNF-α, and MIP-1α) in a concentration-dependent manner in BV2 cells. Gal-9 enhanced neurodegeneration and oxidative stress induced by MPP^+^ in SH-SY5Y cells and primary neurons. Importantly, deletion of Gal-9 or blockade of Tim-3 ameliorated microglial activation, reduced dopaminergic neuronal loss, and improved motor performance in an MPTP-induced mouse model of PD. These observations demonstrate a pathogenic role of the Gal-9/Tim-3 pathway in exacerbating microglial activation, neuroinflammation, oxidative stress, and dopaminergic neurodegeneration in the pathogenesis of PD.

## Introduction

Parkinson’s disease (PD) is one of the most common neurodegenerative diseases. Pathologically, it is characterized by the progressive loss of dopaminergic neurons in the substantia nigra pars compacta (SNpc; Forno, 1996; Surguchov, 2022). Although the pathogenesis of PD remains largely unknown, the intracellular inclusions termed “Lewy bodies” and “Lewy neurites,” mainly consisting of α-synuclein (α-syn), are recognized as the characteristic pathological markers of PD (Polymeropoulos et al., 1997; Lee and Trojanowski, 2006). Neuroinflammation is considered an important pathway that mediates PD pathogenesis (Harry and Kraft, 2008; Lee Y. et al., 2019). Microglial activation, as part of innate immunity, actively participates in neuroinflammation (Imamura et al., 2003). However, the exact molecular mechanisms underlying microglial activation and neurodegeneration in PD need to be further investigated.

Galectins are a group of glycan-binding proteins. They contain highly conserved carbohydrate-recognition domains (CRDs) that interact with β-galactose in glycoconjugates. There are 15 members in the galectin family, which are involved in widespread biological processes, including cell proliferation, migration, adhesion, apoptosis, and endocytosis (Yang et al., 2008). Recent evidence suggests that galectins play a role in the pathogenesis of neurological diseases (Pardo et al., 2019; Siew et al., 2019; Barake et al., 2020). Gal-1 and Gal-8 were found to exert neuroprotective effects in tauopathies and PD (Falcon et al., 2018; Siew and Chern, 2018; Pardo et al., 2019). Gal-1 inhibits microglial activation and reduces pro-inflammatory responses through the MAPK/IκB/NF-κB axis, ameliorating dopaminergic degeneration in MPTP (1-methyl-4-phenyl-1,2,3,6-tetrahydropyridine)-induced PD model (Li et al., 2020). Gal-8 activates autophagic clearance of pathological tau seeds through recruitment of the cargo receptor nuclear dot protein 52 (NDP52), reducing tau pathology (Falcon et al., 2018). Interestingly, Gal-3 acts as a double-edged sword in different neurological diseases (Boza-Serrano et al., 2019; Rahimian et al., 2019; Siew et al., 2019; Jia et al., 2020; Rahimian et al., 2021; Tan et al., 2021). It interacts with triggering receptor expressed on myeloid cells-2 (TREM2), activates microglia, and triggers further Gal-3 expression. In the APP/PS1 transgenic mouse model of Alzheimer’s disease (AD), Gal-3 interacts with amyloid-β and promotes its oligomerization (Tao et al., 2020). Conversely, Gal-3 enhances microglial transformation toward an anti-inflammatory phenotype and decreases infarct size in a mouse model of ischemic stroke, suggesting that Gal3 exerts neuroprotective effects (Rahimian et al., 2019).

Gal-9 is the most highly expressed galectin in the brain (John and Mishra, 2016). It consists of two CRDs connected by a linker sequence. It acts as an immunomodulatory factor in the development of some diseases, such as autoimmune diseases (Panda et al., 2018; Xu et al., 2021), cancer (Klibi et al., 2009; Golden-Mason and Rosen, 2017; Zhang C. X. et al., 2020; Yang et al., 2021), leukemia (Lee et al., 2022), hepatitis (Liberal et al., 2012; Miyakawa et al., 2022), and HIV-1 infection (Elahi et al., 2012). The concentration of Gal-9 in the cerebral spinal fluid (CSF) is elevated in patients with secondary progressive multiple sclerosis and is highly correlated with the number of lesions in the brain (Burman and Svenningsson, 2016). In a rodent model of intracerebral hemorrhage (ICH), the number of M2-type microglia and anti-inflammatory factors was increased along with Gal-9 expression. Gal-9 alleviated brain injury and promoted the recovery of ICH-induced injury (Liang et al., 2021). Furthermore, the levels of Gal-9 are increased in the serum of patients with mild cognitive impairment and AD (Wang et al., 2019). A recent study reported that Gal-9 binds to its receptor, T-cell immunoglobulin and mucin domain 3 (Tim-3), to promote pro-inflammatory phenotype in microglia and increase the release of inflammatory cytokines, leading to neuronal degeneration and aggregating secondary brain injury (Chen et al., 2019). However, it remains unknown whether Gal-9 plays a role in the pathogenesis of PD. Here, we show that microglia-derived Gal-9 promotes neurodegeneration and oxidative stress *in vitro* and *in vivo*. Deletion of Gal-9 or blockade of Tim-3 ameliorates motor deficits and reduces microglial activation in a MPTP-induced PD mouse model.

## Materials and methods

### Mice

Wild-type C57BL/6 and Gal-9 knockout (KO) mice on the C57BL/6 background were obtained from Cyagen Biosciences. Three-month-old male mice were used for intraperitoneal injection. The sample size was determined by Power and Precision (Biostat). Animals were randomly assigned to each group (12 mice per group). Animal care and handling were performed according to the Declaration of Helsinki and the guidelines of Renmin Hospital, Wuhan University. The investigators were blinded to the group assignments. The protocol was reviewed and approved by the Animal Care and Use Committee of Renmin Hospital of Wuhan University.

### Mouse treatment

The MPTP-induced PD model was established as previously reported with slight modifications (Gao et al., 2015; Lee E. et al., 2019). Briefly, WT and Gal-9 KO mice were injected intraperitoneally with 20 mg/kg MPTP (MCE, HY-15608) for 7 consecutive days. The control group was administered with PBS. To investigate the role of Tim-3 in the MPTP-induced PD model, mice were injected intraperitoneally with 50 μg Tim-3 antibody (Proteintech) for seven consecutive days 2 h before MPTP/PBS administration. The dosage and treatment methods were based on previous studies with slight modification (Koyama et al., 2016; Dixon et al., 2021). The behavioral deficit was evaluated 24 h after the final PBS or MPTP injection. Then, the mice were sacrificed for extraction of the brain tissues.

### Behavioral tests

Motor tests were evaluated 24 h after the final PBS or MPTP injection, including the pole test, balance beam test, and rotarod test. The mice were transported to the experimental room 30 min before behavioral tests for acclimatization. In the pole test, mice were placed on the top of a vertical wooden pole (50 cm long with 1 cm diameter) with a rough surface and performed an autonomous descending. Mice were trained twice before test. The time to reach the base of the pole with their paws was recorded. In the balance beam test, mice were trained to walk across a wooden beam (17 mm in width and 80 cm in length) with a cube at the terminus. One day after training, the test was performed on a beam with a relatively narrow width (10 mm). The time to reach the terminus was collected. The test was performed 3 times with 10 min intervals. The average time was recorded for each mouse. In the rotarod test, mice were initially trained for 2 min at a speed of 4 r.p.m. During the tests, the mice were placed on the rotarod rod with a gradually increasing speed from 4 r.p.m. to 40 r.p.m. The maximum cutoff time to stop the test and recording was 200 s. The fall-off time was recorded.

### RT-PCR

To measure the levels of mRNA, total RNA was isolated from cultured cells or mouse brain tissue using TRIzol reagent (Invitrogen) and reverse transcribed to complementary DNA using the iScript cDNA synthesis kit (Bio-Rad). Quantitative real-time PCR (RT-PCR) was performed using Light Cycler 480 SYBR Green 1 Master Mix (Roche). The primers are listed in Table 1. The relative expression levels of target proteins were normalized to that of GAPDH and assessed using the 2^−ΔΔCt^ method.

**Table 1:** A list of primers for RT-PCR analysis.

Gene	Forward	Reverse
m-IL-6	TAGTCCTTCCTACCCCAATTTCC	TTGGTCCTTAGCCACTCCTTC
m-IL-1β	GAAATGCCACCTTTTGACAGTG	TGGATGCTCTCATCAGGACAG
m-TNF-α	CCCTCACACTCAGATCATCTTCT	GCTACGACGTGGGCTACAG
m-MIP-1α	TTCTCTGTACCATGACACTCTGC	CGTGGAATCTTCCGGCTGTAG
m-Arg-1	CTCCAAGCCAAAGTCCTTAGAG	AGGAGCTGTCATTAGGGACATC
m-GAPDH	AGGTCGGTGTGAACGGATTTG	TGTAGACCATGTAGTTGAGGTCA

### Enzyme-linked immunosorbent assay

BV2 cells were treated with 500 μM MPP+, the dose was chosen in reference to previous studies (Liu et al., 2020; Zheng et al., 2021). The culture medium of BV2 cells treated with PBS or MPP^+^ for 24 h was harvested. The concentrations of Gal-9 in the medium were measured by a sandwich Enzyme-linked immunosorbent assay (ELISA) kit (Mlbio, ml558358-C). In brief, the 96-well microtiter plate was pre-coated with the Gal-9 antibody. The plate was loaded with 50 μl samples and 100 μl HRP-conjugated detection antibody in each well. After incubation for 1 h at 37°C, the plate was washed five times with washing buffer. Then, 100 μl of 3,3′,5,5′-tetramethylbenzidine (TMB) substrate was added to each well and incubated for 15 min in the dark. The reaction was terminated by adding 50 μl/well of stop solution. Signals were measured on a microplate reader at 450 nm (Molecular Devices).

### Western blot

The cells and mouse brain tissue were homogenized in ice-cold lysis buffer (50 mM Tris, pH 7.4, 40 mM NaCl, 1 mM EDTA, 0.5% Triton X-100, 1.5 mM Na3VO4, 50 mM NaF, 10 mM sodium pyrophosphate, and 10 mM sodium β-glycerophosphate) supplemented with phosphatase inhibitor mixture and cocktail (Roche) for 45 min. Then, the samples were centrifuged at 21,130 g for 30 min at 4°C. The protein concentrations were determined by Pierce BCA Protein Assay Kit (Thermo Fisher). Samples were separated by 10% SDS-polyacrylamide gels and transferred to nitrocellulose membranes. The membranes were blocked with 5% non-fat milk in TBS containing 0.1% Tween 20 (TBST) and then incubated with primary antibody overnight at 4°C. The membranes were washed 3 times with TBST and incubated with appropriate HRP-conjugated secondary antibodies (BIO-RAD, 1:3,000) for 1 h at room temperature. Finally, the bands were visualized using ECL substrates in the ChemiDoc Gel Imaging System (Bio-Rad). Primary antibodies included Gal-9 (Abcam, ab227046, 1:1,000), Tim-3 (Abcam, ab252533, 1:1,000), caspase-1 (ABclonal, A0964, 1:1,000), IL-1β (ABclonal, A17361, 1:1,000), cleaved IL-1β (Affinity, AF4006, 1:1,000), TH (Abcam, ab112, 1:1,000), IBA1 (Proteintech, 10,904-1-AP, 1:800), GFAP (Proteintech, 60,190-1-Ig, 1:1,000), and GAPDH (Proteintech, 60,004-1-Ig, 1:5,000).

### Cell viability assay

Cell viability was determined in SH-SY5Y cells treated with PBS, Gal-9 (16 nM, AtaGenix), MPP^+^ (500 μM, MCE), or MPP^+^ + Gal-9. The cells were seeded into 96-well plates at a density of 8,000 cells per well. After treatments, the cells were incubated with 100 μl fresh medium with 10 μl CCK-8 solution (Abbkine, BMU106-CN) for 2 h at 37°C. The optical density (OD) was measured at 450 nm by a microplate reader (Molecular Devices).

### Primary neuronal culture

Primary cortical neurons derived from WT mice at embryonic day 18 were cultured as previously described (Zhang et al., 2014, 2022). In brief, the neurons were cultured in neurobasal media supplemented with L-glutamine and B27 (Invitrogen, Carlsbad, CA). On 6 days *in vitro* (DIV), the neurons were treated for 12 h with PBS, MPP^+^, Gal-9, MPP^+^ + Gal-9, or conditioned medium (CM) from BV2 cells for 24 h. To delete Gal-9, the conditioned medium of MPP^+^-treated BV2 cells was incubated with Gal-9 antibody (Proteintech, Cat No. 17938-1-AP, 1:300) and protein A/G beads overnight at 4°C. After treatment, the neurons were fixed in 4% paraformaldehyde (PFA) for 30 min, permeabilized in 4% PFA containing 1% Triton X-100 for 10 min, and blocked and immunostained with MAP 2 antibody (Proteintech, 17,490–1-AP, 1:1,000) overnight at 4°C. The neurotoxic effect of MPP^+^ and Gal-9 was determined by a TUNEL BrightRed Apoptosis Detection Kit (Vazyme).

### TUNEL assay

SH-SY5Y cells and primary cortical neurons were fixed with 4% PFA and incubated in PBS containing 1% Triton X-100 for 5 min. After being washed 3 times with PBS, the slides were labeled with terminal deoxynucleotidyl transferase (TdT) reaction buffer (recombinant TdT enzyme, bright red labeling mix, equilibration buffer, ddH_2_O) for 1 h at 37°C. For primary cortical neurons, the slides were immunostained with MAP 2 antibody overnight followed by incubation with 488-conjugated secondary antibodies. Cell nuclei were counterstained with 4′,6-diamidino-2-phenylindole (DAPI).

### Measurement of oxidative stress

To detect the levels of reactive oxygen species (ROS), DCFH-DA (Beyotime, S0033S) was used as the probe to measure the intracellular ROS levels in SH-SY5Y cells after exposure to MPP^+^ with or without Gal-9 for 24 h. Briefly, the cells were washed with PBS 3 times and incubated with DCFH-DA (10 μM) dissolved in serum-free medium for 20 min at 37°C. The fluorescence intensity was examined by a microplate reader at excitation and emission wavelengths of 488 and 525 nm, respectively. To determine the levels of hydrogen peroxide (H_2_O_2_), mouse brain tissue and SH-SY5Y cells were homogenized in ice-cold acetone. The samples were centrifuged at 8,000 ×g for 10 min. The concentration of H_2_O_2_ in the supernatant was determined according to the protocol of the Hydrogen Peroxide Assay Kit (Solarbio, Cat# BC3595). To measure the level of MDA and the activity of GPx and CAT, mouse brain tissue and SH-SY5Y cells were lysed in lysis buffer. After centrifugation at 10,000 ×g for 10 min at 4°C, the supernatant of the samples was collected for the analysis of CAT activity (Beyotime, S0051), GPx activity (Beyotime, S0056), and MDA levels (Beyotime, S0131S) following the manufacturer’s instructions.

### Caspase-9 assay

BV2 cells were exposed to 500 μM MPP^+^ for 24 h. The enzymatic activity of caspase-9 was examined using a Caspase-9 Activity Assay kit (C1157, Beyotime, China). Briefly, 50 μl cell lysate, 40 μl caspase-9 reaction buffer, and 10 μl caspase substrate (Ac-DEVD-pNA) were added to a 96-well plate. After incubation at 37°C for 2 h, the samples were read with a microplate reader (Molecular Devices) at 405 nm.

### Immunofluorescence

Anesthetized mice were received cardiac perfusion with PBS and 4% PFA. The brains were removed and postfixed in 4% PFA overnight and then in 30% sucrose. Next, serial brain sections sliced to 20 μm thickness were processed with 1% Triton X-100 in PBS, blocked with 3% BSA, and incubated with primary antibodies against TH (Abcam, ab112, 1:1,000) and IBA1 (Wako, 019–19,741, 1:1,000) overnight at 4°C. Then, the brain sections were incubated with Alexa Fluor 594- and 488-conjugated secondary antibodies (Thermo Fisher, 1:700). The signals were detected by fluorescence microscopy (Olympus IX73).

### Statistical analysis

All data are shown as means ± SEM of at least 3 independent experiments. Statistical analysis was performed with GraphPad Prism 7.0. Statistical comparison between two groups was analyzed by unpaired two-tailed Student’s *t*-test. One-way ANOVA and two-way ANOVA were used to analyze data from more than two groups followed by Tukey’s *post-hoc* tests. The statistical significance was determined by a *p-*value < 0.05.

## Results

### MPP^+^ induces microglial activation and Gal-9 expression

**Figure 1:**
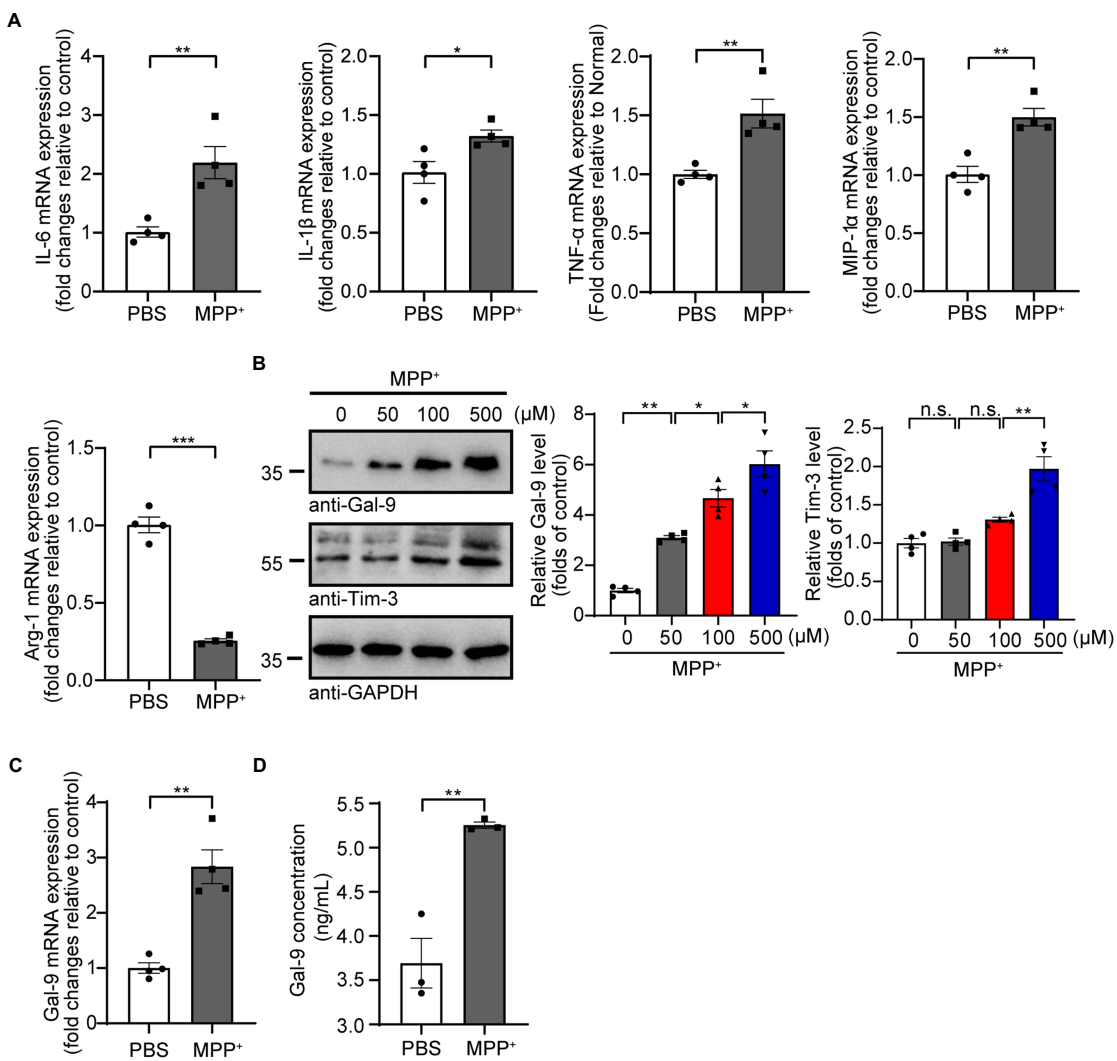
MPP^+^ promotes microglial activation and Gal-9 expression. **(A)** The mRNA levels of IL-6, IL-1β, TNF-α, MIP-1α, and Arg-1 were measured by RT-PCR in BV2 cells treated with MPP^+^ (500 μM) for 24 h. Data are presented as the means ± SEM. Unpaired Student’s *t*-test (*n* = 4 independent experiments). **(B)** Representative immunoblots and quantification of Gal-9 and Tim-3 in lysates from BV2 cells treated with different concentrations of MPP^+^ (0, 50, 100, and 500 μM) for 48 h. Data are presented as means ± SEM. One-way ANOVA followed by Tukey’s *post-hoc* test (*n* = 4). **(C)** The mRNA levels of Gal-9 in BV2 cells were assessed by RT-PCR after treatment with MPP^+^ (500 μM) for 24 h. Data are presented as means ± SEM. Unpaired Student’s *t*-test (*n* = 4). **(D)** Quantification of Gal-9 levels in the culture medium of MPP^+^-treated BV2 cells by ELISA. Data are presented as the means ± SEM. Unpaired Student’s *t*-test (*n* = 3). ^*^p < 0.05, ^**^p < 0.005, ^***^p < 0.0005.

We first investigated the effect of MPP^+^ on the expression of pro-inflammatory cytokines (IL-6, IL-1β, TNF-α, and MIP-1α) and an anti-inflammatory cytokine (Arg-1) in BV2 microglial cells. RT-PCR analysis found that the mRNA levels of pro-inflammatory cytokines in BV2 cells were increased, whereas the mRNA level of the anti-inflammatory cytokine Arg-1 was reduced after MPP^+^ treatment (Figure 1A), indicating that MPP^+^ induces the pro-inflammatory activation of microglia. Interestingly, MPP^+^ treatment increased the levels of pro-IL-1β but not mature IL-1β or caspase-1 in BV2 cells (Supplementary Figures 1A,B). These results are consistent with previous reports (Lee E. et al., 2019; Zheng et al., 2021). In addition, the caspase-9 activity of BV2 cells was increased after MPP^+^ treatment (Supplementary Figure 1C), suggesting that MPP^+^ induces the apoptosis of BV2 cells. Subsequently, we investigated whether MPP^+^-mediated microglial activation is accompanied by elevated Gal-9 expression. As expected, the expression of Gal-9 and its receptor Tim-3 was increased in BV2 cells after MPP^+^ treatment in a concentration-dependent manner (Figure 1B). The level of Gal-9 mRNA was also increased after MPP^+^ treatment (Figure 1C). Furthermore, we performed a sensitive enzyme-linked immunosorbent assay (ELISA) to determine the concentrations of Gal-9 in the conditioned medium (CM) of BV2 cultures after MPP^+^ treatment and found that MPP^+^ increased the levels of Gal-9 in the CM. These results indicate that MPP^+^ promotes the expression and secretion of Gal-9 by microglia (Figure 1D).

### Gal-9 enhances the toxic effect of MPP^+^ in SH-SY5Y cells and primary neurons

**Figure 2:**
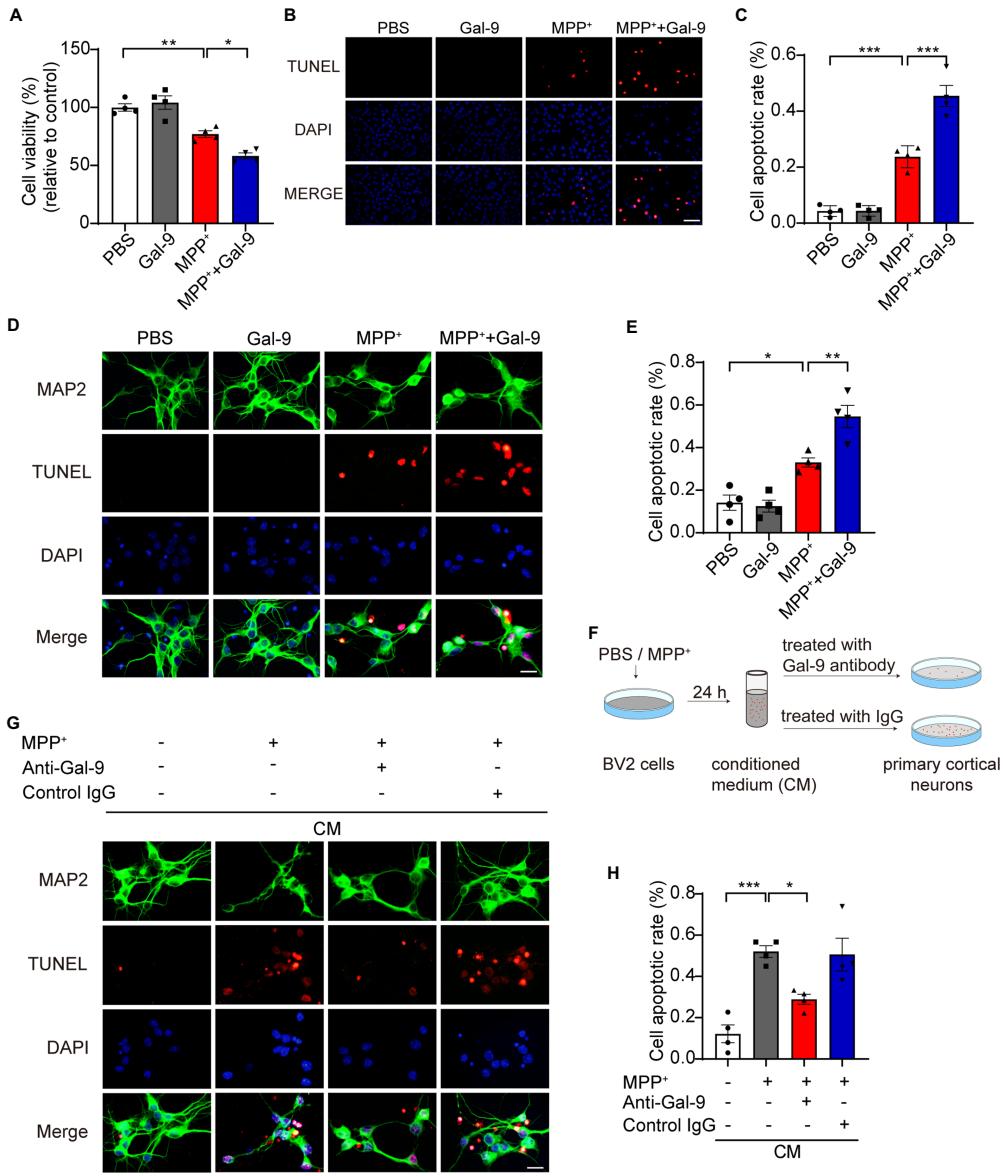
Gal-9 enhances neurotoxicity induced by MPP^+^ *in vitro*. **(A)** Cell viability was measured by CCK-8 assay in SH-SY5Y cells treated with PBS, Gal-9 (16 nM, 24 h), MPP^+^ (500 μM, 24 h), and MPP^+^ together with Gal-9. Data are presented as means ± SEM. One-way ANOVA followed by Tukey’s *post-hoc* test (*n* = 4 independent experiments). **(B,C)** Representative immunofluorescence images **(B)** and quantification **(C)** of apoptotic SH-SY5Y cells by TUNEL assay. Scale bar, 20 μm. Data are presented as means ± SEM. One-way ANOVA followed by Tukey’s *post-hoc* test (*n* = 4 independent experiments). **(D,E)** Representative images **(D)** and quantification **(E)** of TUNEL staining of primary cortical neurons treated with MPP^+^ (100 μM) and Gal-9 (16 nM) for 12 h. Scale bar, 20 μm. Data are presented as means ± SEM. One-way ANOVA followed by Tukey’s *post-hoc* test (*s* = 4 independent experiments). **(F)** Schematic for transferring conditioned medium into primary neurons. **(G)** The apoptosis of primary neurons cultured for 24 h with conditioned medium was detected by TUNEL assay. Scale bar, 20 μm. **(H)** Quantification of apoptotic primary cortical neurons cultured with conditioned medium. Data are presented as means ± SEM. One-way ANOVA followed by Tukey’s *post-hoc* test (*n* = 4 independent experiments). ^*^p < 0.05, ^**^p < 0.005, ^***^p < 0.0005.

To investigate whether Gal-9 is involved in MPP^+^-induced neurotoxicity, a CCK-8 assay was used to assess the survival of SH-SY5Y cells exposed to MPP^+^ in the presence or absence of Gal-9 (Figure 2). As expected, we found a significant reduction in cell viability when the cells were exposed to MPP^+^. Gal-9 alone did not alter cell viability but exacerbated the toxic effect of MPP^+^ (Figure 2A). Apoptotic cells were detected by TUNEL assay after MPP^+^ treatment. Similar to the CCK-8 assay results, Gal-9 increased the percentage of apoptotic cells induced by MPP^+^ (Figures 2B,C). We further verified the effect of Gal-9 in cultured primary cortical neurons by TUNEL assay. Similar to the observations in SH-SY5Y cells, Gal-9 increased the percentage of apoptotic cells induced by MPP^+^ (Figures 2D,E).

Given that neurodegeneration is closely related to microglia-mediated neuroinflammation, we further tested whether Gal-9 mediates microglia-mediated neurotoxicity. BV2 cells were pre-treated with MPP^+^ for 24 h. The CM was collected and transferred into primary neurons. As expected, the CM from MPP^+^-treated BV2 cells induced apoptosis of primary neurons. Interestingly, deletion of Gal-9 *via* antibody from the CM dramatically attenuated the toxic effect of microglial CM (Figures 2G,H). These findings indicate that Gal-9 is required for the toxic effect of CM from MPP^+^-treated BV2 cells.

### Gal-9 exacerbates MPP^+^-induced oxidative stress in SH-SY5Y cells

**Figure 3:**
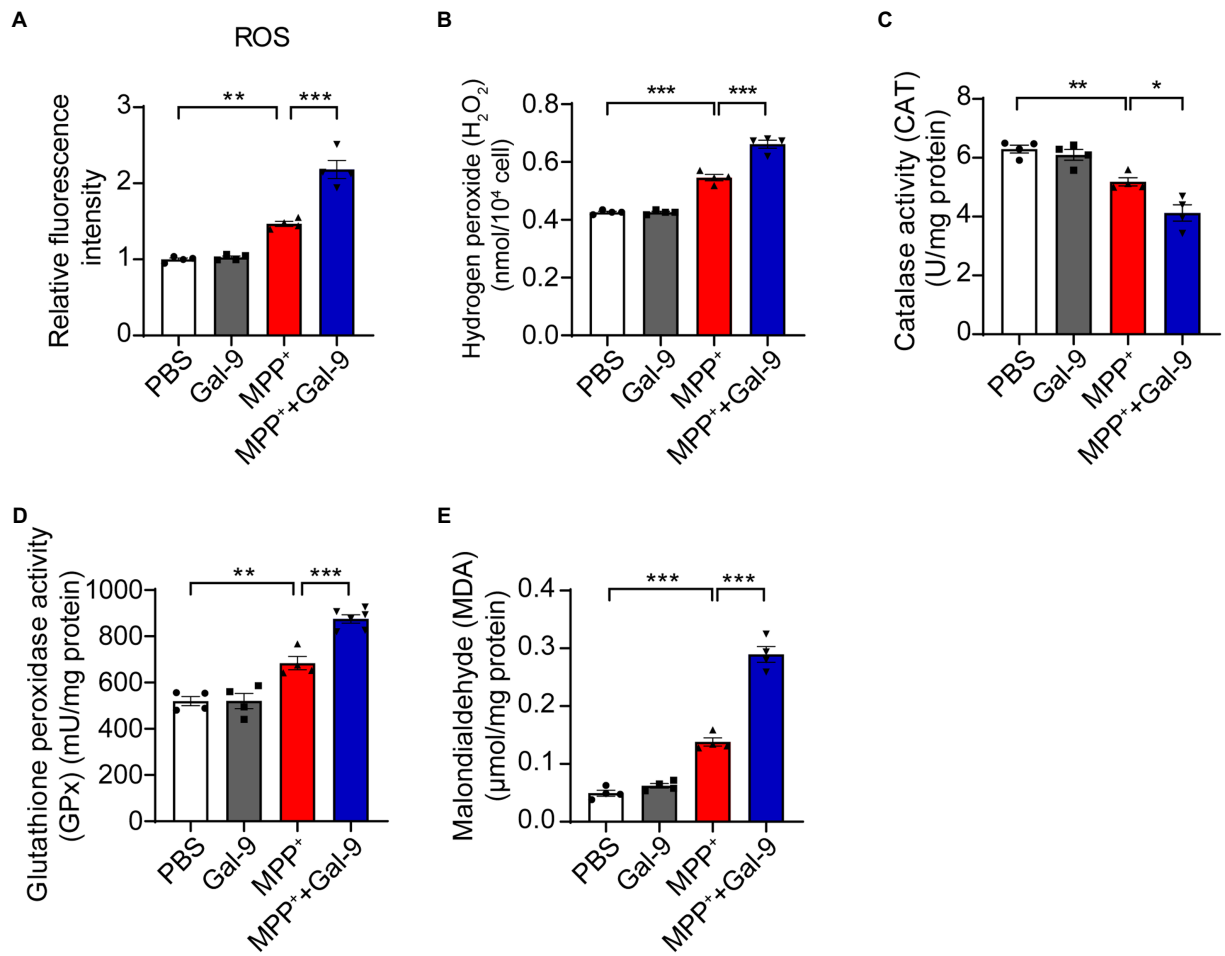
Gal-9 exacerbates oxidative stress induced by MPP^+^. **(A,B)** The levels of ROS and H_2_O_2_ in SH-SY5Y cells exposed to MPP^+^ (500 μM) and Gal-9 (16 nM) for 24 h. *n* = 4 independent experiments. **(C,D)** The activity of catalase (CAT) and glutathione peroxidase (GPx) in SH-SY5Y cells exposed to MPP^+^ or MPP^+^ plus Gal-9 (*n* = 4 independent experiments). **(E)** The levels of malondialdehyde (MDA) in SH-SY5Y cells exposed to MPP^+^ and Gal-9. Data are presented as means ± SEM. One-way ANOVA followed by Tukey’s *post-hoc* test (*n* = 4 independent experiments). ^*^p < 0.05, ^**^p < 0.005, ^***^p < 0.0005.

MPP^+^ induces mitochondrial dysfunction and promotes oxidative stress (Nagatsu and Sawada, 2006; Chia et al., 2020). To investigate whether Gal-9 plays a role in MPP^+^-induced oxidative stress, we tested the accumulation of reactive oxygen species (ROS) using DCFH-DA probes in SH-SY5Y cells after treatment with MPP^+^ in the presence or absence of Gal-9. MPP^+^ promoted the production of ROS, which was further increased in the presence of Gal-9 (Figure 3A). It is likely that the levels of hydrogen peroxide (H_2_O_2_) were also increased in the presence of MPP^+^ and further enhanced by Gal-9 (Figure 3B).

In accord with these observations, we observed a marked reduction in antioxidant enzyme activity, such as catalase (CAT) and glutathione peroxidase (GPx), in MPP^+^-treated SH-SY5Y cells compared to cells treated with PBS or Gal-9 alone. The reduction in CAT and GPx was further aggravated by Gal-9 (Figures 3C,D). Furthermore, the elevation of malondialdehyde (MDA, a lipid oxidative product) in SH-SY5Y cells exposed to MPP^+^ + Gal-9 also confirmed the promotive effect of Gal-9 on MPP^+^-induced oxidative stress (Figure 3E). Collectively, Gal-9 alone does not induce ROS, but exacerbates oxidative stress induced by MPP^+^.

### Gal-9 knockout or Tim-3 blockade ameliorates dopaminergic neurodegeneration induced by MPTP *in vivo*

**Figure 4:**
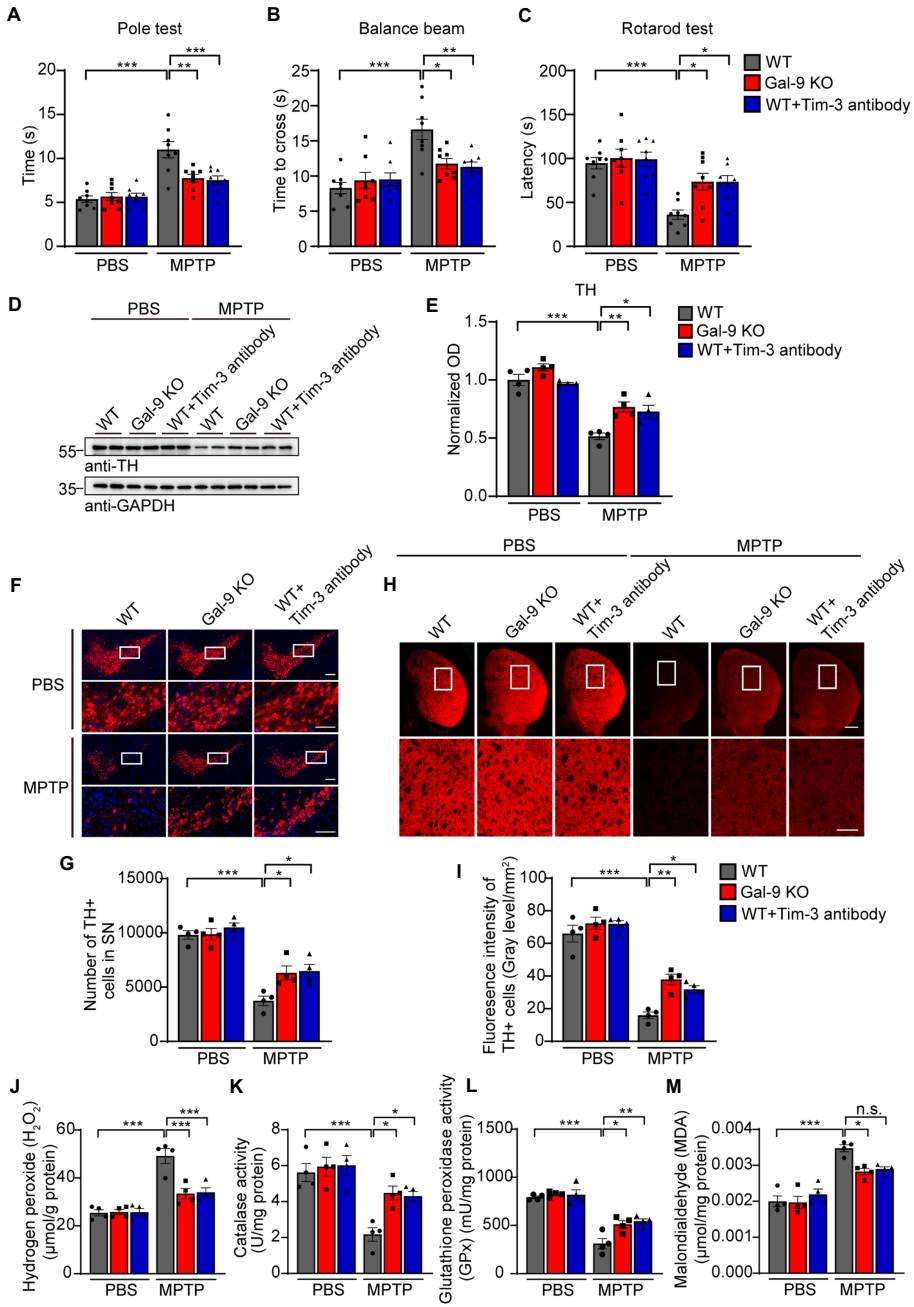
Blockade of the Gal-9/Tim-3 pathway ameliorates motor dysfunction and dopaminergic neuronal loss in MPTP-treated mice. **(A–C)** The pole test, balance beam test, and rotation test were performed in WT, Gal-9 KO, and WT mice pre-administered with Tim-3 antibody. Data are presented as means ± SEM. Two-way ANOVA followed by Tukey’s *post-hoc* test (*n* = 8 mice per group). **(D,E)** Representative immunoblots **(D)** and quantification **(E)** of TH in SN lysates from WT, Gal-9 KO, and WT mice pre-administered with Tim-3 antibody. Data are presented as means ± SEM. Two-way ANOVA followed by Tukey’s *post-hoc* test (*n* = 4 mice per group). **(F)** Representative immunostaining of TH in the SNpc of WT, Gal-9 KO, and WT mice pre-administered with Tim-3 antibody. Scale bars, 200 μm for upper panel, 100 μm for magnification. **(G)** Stereological counts of TH^+^ neurons in the SNpc. Data are presented as means ± SEM. Two-way ANOVA followed by Tukey’s *post-hoc* test (*n* = 4 mice per group). **(H)** Representative TH staining in the striatum. Scale bars, 500 μm for upper panel, 200 μm for magnification. **(I)** Quantification of TH immunoreactivity in the striatum. Data are presented as means ± SEM. Two-way ANOVA followed by Tukey’s *post-hoc* test (*n* = 4 mice per group). **(J–M)** The levels of H_2_O_2_, CAT, GPx, and MDA in the SN of WT, Gal-9 KO, and WT mice pre-administered with Tim-3 antibody after PBS or MPTP treatment. Data are presented as means ± SEM. Two-way ANOVA followed by Tukey’s *post hoc* test (*n* = 4 mice per group). ^*^p < 0.05, ^**^p < 0.005, ^***^p < 0.0005.

To investigate whether Gal-9 is involved in PD-related neurodegeneration *in vivo*, we chose a classical PD model by intraperitoneal injection of MPTP (20 mg/kg body weight) for 7 days (Figure 4). We compared neurodegeneration induced by MPTP in the wild-type and Gal-9 knockout mice. To assess the role of the Gal-9 receptor Tim-3 in the PD model, WT mice were intraperitoneally injected with Tim-3 antibody (50 μg/mice) for 7 days 2 h before MPTP injection to block Tim-3 function. A panel of behavioral tests was performed to assess the motor function of PD mice, including the pole test, balance beam test, and rotarod test (Figures 4A–C). MPTP treatment caused deficits in WT mice in the pole test, a sensitive examination of motor function, whereas the deficits were markedly ameliorated in Gal-9 KO mice and in mice pre-treated with Tim-3 antibody. It is likely that MPTP treatment induced prolonged latency on the balance beam test in WT mice, which was attenuated by Gal-9 KO or Tim-3 blockade. Together, these behavioral tests indicated that Gal-9 KO or Tim-3 blockade could reduce the motor impairment induced by MPTP.

Next, we investigated the effect of the Gal-9/Tim-3 pathway on the degeneration of dopaminergic neurons. Immunoblotting showed that MPTP treatment induced a significant reduction in tyrosine hydroxylase (TH) levels in the SNpc of WT mice. This reduction was significantly alleviated in Gal-9 KO mice and mice treated with Tim-3 antibody (Figures 4D,E). Immunofluorescence demonstrated that MPTP treatment induced a dramatic reduction in TH-positive neurons in the SN of WT mice, which was attenuated by Gal-9 KO or Tim-3 blockade (Figures 4F,G). Furthermore, MPTP-treated mice exhibited weak TH immunoreactivity in the striatum compared with the control mice, while Gal-9 KO or Tim-3 blockade ameliorated the poor performance (Figures 4H,I).

To explore the effect of Gal-9 or Tim-3 on mitochondrial dysfunction and oxidative stress in PD mice, we further examined the levels of H_2_O_2_ and malondialdehyde and the activity of endogenous antioxidant enzymes, including CAT and GPx, in the SN (Figures 4J–M). MPTP caused high H_2_O_2_ and malondialdehyde levels in WT mice, which were attenuated in Gal-9 KO or Tim-3 blockade mice. Likewise, compared with WT mice, MPTP administration induced decreased activity of catalase and glutathione peroxide, demonstrating a higher level of oxidative stress. Thus, these findings demonstrated that the Gal-9/Tim-3 pathway is involved in MPTP-induced loss of dopaminergic neurons, oxidative stress, and motor impairments.

### Blockade of the Gal-9/Tim-3 pathway attenuates microglial activation in MPTP-treated mice

**Figure 5:**
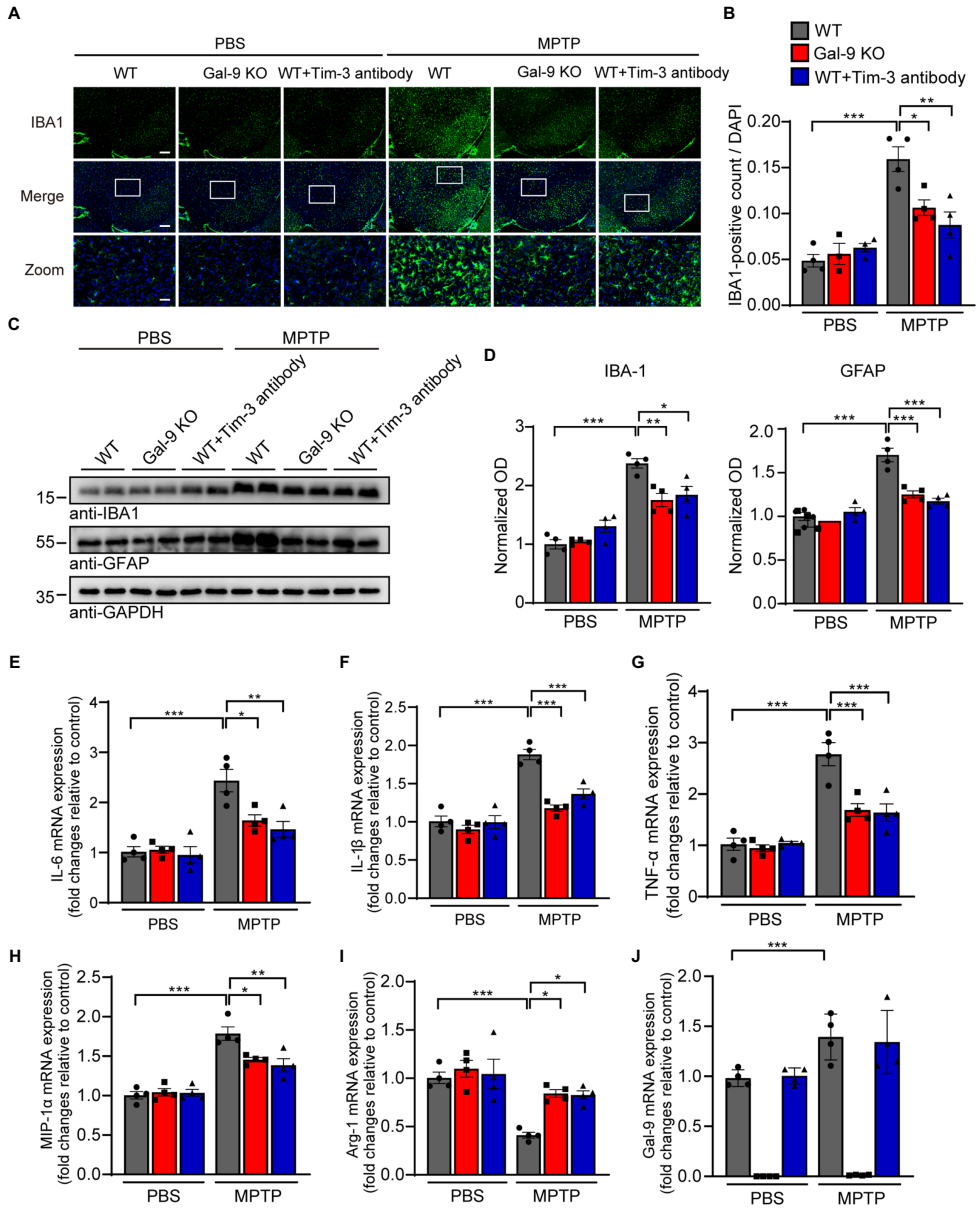
Blockade of the Gal-9/Tim-3 pathway attenuates neuroinflammation in MPTP-induced PD mice. **(A,B)** Representative images **(A)** and quantification **(B)** of the microglial marker IBA1 in the SN area. Scale bar, 200 μm in the upper panel, 50 μm in magnified pictures. Data are presented as means ± SEM. Two-way ANOVA followed by Tukey’s *post-hoc* test (*n* = 4 mice per group). **(C,D)** Representative immunostaining **(C)** and quantification **(D)** of the microglial marker IBA1 and astrocytic marker GFAP in WT, Gal-9 KO, and WT mice pre-administered with Tim-3 antibody after PBS or MPTP treatment. Data are presented as means ± SEM. Two-way ANOVA followed by Tukey’s *post-hoc* test (*n* = 4). **(E–J)** The mRNA levels of IL-6, IL-1β, TNF-α, MIP-1α, Arg-1, and Gal-9 in the SN were measured by RT–PCR. *n* = 4 mice per group. Data are presented as means ± SEM. Two-way ANOVA followed by Tukey’s *post-hoc* test. ^*^p < 0.05, ^**^p < 0.005, ^***^p < 0.0005.

Since neuroinflammation is widely recognized to contribute to neurodegeneration, we sought to investigate whether Gal-9 KO or Tim-3 blockade was correlated with microglial activation in MPTP-treated mice. Interestingly, immunofluorescence indicated that MPTP administration induced a considerable increase in microglial activation in the SN of WT mice. However, there were relatively few activated microglia in Gal-9 KO and Tim-3 blockade mice (Figures 5A,B). Immunoblotting for IBA1 (a microglial marker) confirmed the elevated expression of microglia in WT mice (Figures 5C,D). Furthermore, we examined the expression of the astrocytic marker GFAP and found a similar expression pattern to microglia (Figures 5C,D). We further assessed the mRNA levels of inflammatory cytokines and gal-9 in the SN (Figures 5E–J). RT-PCR assay showed that the pro-inflammatory cytokines, such as IL-6, IL-1β, TNF-α, and MIP-1α, were increased in MPTP-treated WT mice. However, Gal-9 KO and Tim-3 blockade reduced the expression of pro-inflammatory cytokines in MPTP-treated mice. In contrast, the anti-inflammatory cytokine Arg-1 was decreased in MPTP-treated WT mice, and this decrease was reversed by Gal-9 KO and Tim-3 blockade. These data suggest that blockade of the Gal-9/Tim-3 pathway attenuates MPTP-induced neuroinflammation in the SN.

## Discussion

As an evolutionarily conserved glycan-binding protein, Gal-9 acts as an endogenous modulator of neuroinflammation. In the present study, we show that the Gal-9/Tim-3 pathway is involved in MPTP-induced neurodegeneration *in vitro* and *in vivo*. Specifically, blockade of the Gal-9/Tim-3 pathway reduces microglial activation, attenuates mitochondrial oxidative stress, and prevents neurodegeneration and behavioral deficits induced by MPTP injection. These results support that the Gal-9/Tim-3 pathway might play an important role in the pathogenesis of PD by regulating neuroinflammation and mitochondrial oxidative stress.

As a member of the galectin family, Gal-9 is involved in various physiological and pathological processes, including cell adhesion (Eckardt et al., 2020; Krautter and Iqbal, 2021; Iqbal et al., 2022; Pally et al., 2022), migration (Mansour et al., 2022), differentiation (Fan et al., 2018; Bertino et al., 2019), angiogenesis (Aanhane et al., 2018; Hill et al., 2021), and immunomodulation (Fan et al., 2018; Wiersma et al., 2019; Yan et al., 2022). In the central nervous system, Gal-9 is mainly expressed in microglia (Steelman et al., 2013). A recent study demonstrated the roles of the Gal-9/Tim-3 pathway in promoting the production of pro-inflammatory factors and in aggravating brain injury induced by intracerebral hemorrhage (Chen et al., 2019). Recombinant Gal-9 promoted the expression of pro-inflammatory cytokines by microglia (Steelman and Li, 2014). In our study, we found that MPP^+^ elevated the expression of Gal-9 and Tim-3 in BV2 microglial cells in a concentration-dependent manner. Consistent with previous reports (Liu et al., 2020; Zhang Y. et al., 2020), we found that MPP^+^ promoted the expression of pro-inflammatory cytokines in BV2 cells, including TNF-α, IL-6, IL-1β, and MIP-1α, whereas it decreased the expression of the anti-inflammatory cytokine Arg-1, suggesting that MPP^+^ induces microglial activation and transforms microglia into pro-inflammatory phenotype. A recent study showed that Gal-1 protects the apoptosis of SH-SY5Y cells induced by MPP^+^ (Liu et al., 2022). We showed that Gal-9 exacerbated MPP^+^-induced apoptosis, as assessed by CCK-8 and TUNEL assays. These results support the functional differences in galectin family members. Furthermore, the CM from MPP^+^-treated BV2 cells induced apoptosis of primary cortical neurons, while depletion of Gal-9 from the CM attenuated the toxic effect of the conditioned medium. These results support the key role of Gal-9 in mediating neurotoxicity. Furthermore, we sought to identify the specific mechanism underlying potent neuronal apoptosis promoted by Gal-9.

MPP^+^ has been shown to be taken up by dopaminergic neurons *via* dopamine transporters and induces mitochondrial oxidative stress and dopaminergic neurodegeneration (Subramaniam and Chesselet, 2013; Martí et al., 2017; Ren et al., 2019). Interestingly, we found that Gal-9 exacerbates MPP^+^-induced intracellular ROS levels and H_2_O_2_ production, decreased antioxidant enzyme activities, and increased lipid oxidation. In MPTP-induced PD mouse model, Gal-9 KO alleviated MPTP-induced loss of dopaminergic neurons, attenuated mitochondrial oxidative stress, and improved motor function. Therefore, we assume that upregulation of Gal-9 may exacerbate mitochondrial oxidative stress in dopaminergic neurons, thus leading to dopaminergic neurodegeneration and motor deficits. It is noteworthy that functional blockade of Tim-3, the receptor of Gal-9, by intraperitoneal injection of Tim-3-antibody exerted similar effects to Gal-9 KO in MPTP-treated PD mice. Tim-3 has been shown to be highly expressed in microglia that can upregulate Gal-9 (Anderson et al., 2007; Chen et al., 2019). Therefore, the Gal-9/Tim-3 pathway is involved in MPTP-induced dopaminergic neurodegeneration.

Galectins have been reported to contribute to neurodegeneration by participating in neuroinflammation (Barake et al., 2020; da Rosa et al., 2021; Rahimian et al., 2021; Ge et al., 2022). Gal-3, a critical regulator of innate immunity in the brain, is deemed crucial for resident microglial activation and has been shown to play a role in AD pathology (Boza-Serrano et al., 2019; Tan et al., 2021), while deletion of Gal-3 in 5xFAD transgenic mice attenuated microglia-associated immune responses (Boza-Serrano et al., 2019). Notably, Gal-1 was recently demonstrated to inhibit microglial activation and ameliorate neurodegenerative processes in PD and aged mice through its carbohydrate-recognition domain (Li et al., 2020; Shen et al., 2021). Elevated expression of Gal-9 in the cerebrospinal fluid was related to immune activation of the central nervous system and was correlated with poor cognition in HIV-infected older adults (Premeaux et al., 2019). Here, we show that MPP^+^ induced the release of Gal-9 and pro-inflammatory cytokines. Blockade of the Gal-9/Tim-3 pathway suppressed microglial activation and reduced pro-inflammatory cytokine release in the SN of MPTP-induced PD mice, suggesting an important role of the Gal-9/Tim-3 pathway in PD pathology through microglia-dominated neuroinflammation.

In conclusion, we provide evidence revealing a previously unknown role of microglial Gal-9 in PD pathogenesis. The Gal-9/Tim-3 pathway enhances neuroinflammation and exacerbates the toxic effect of MPP^+^. Thus, the Gal-9/Tim-3 pathway may be a novel therapeutic target for PD. New therapeutic strategies targeting the Gal-9/Tim-3 pathway include competitive carbohydrates, small non-carbohydrate binding molecules, and neutralizing antibodies. Several polysaccharides, galactomannan oligomers, and oligopeptides have been found to regulate the activity of the galectin family (Wdowiak et al., 2018). Most recently, it was reported that Gal-9-neutralizing antibodies efficiently protect human T cells from Gal-9-induced cell death (Yang et al., 2022). It is conceivable that Gal-9-targeted therapeutics hold promise for the treatment of PD.

## Data availability statement

The original contributions presented in the study are included in the article/Supplementary material, further inquiries can be directed to the corresponding authors.

## Ethics statement

The animal study was reviewed and approved by the Animal Care and Use Committee of Renmin Hospital of Wuhan University.

## Author contributions

GZ worked on the experimental design and completed the animal modeling. QP and XG conducted the experiments. QP carried out all data analyses and drafted the original manuscript. LD and MX offered technical assistance. ZhaZ, LC, and ZheZ reviewed and contributed to the final version. All authors contributed to the article and approved the submitted version.

## Funding

This work was supported by grants from the National Key Research & Development Program of China (2019YFE0115900), the National Natural Science Foundation of China (nos. 81771382 and 81822016), and the Medical Science Advancement Program of Wuhan University, grant (TFLC2018001).

## Conflict of interest

The authors declare that the research was conducted in the absence of any commercial or financial relationships that could be construed as a potential conflict of interest.

## Publisher’s note

All claims expressed in this article are solely those of the authors and do not necessarily represent those of their affiliated organizations, or those of the publisher, the editors and the reviewers. Any product that may be evaluated in this article, or claim that may be made by its manufacturer, is not guaranteed or endorsed by the publisher.

## Supplementary material

SUPPLEMENTARY FIGURE 1 MPP^+^ induces increased expression of pro-IL-1β in BV2 microglial cells. **(A)** Representative immunoblots of BV2 cells treated with MPP^+^ (500 μM, 24 h). The cellular lysates were immunoblotted with the indicated antibodies. **(B)** Quantification of pro-IL-1β in the cellular lysates of BV2 cells treated with MPP^+^. Data are presented as means ± SEM. Unpaired Student’s *t*-test (n = 3 independent experiments). **(D)** Caspase-9 activity in BV2 cells treated with MPP^+^ (500 μM, 24 h). Data are presented as means ± SEM. Unpaired Student’s *t* test (n = 3). **p* < 0.05, ***p* < 0.005, ****p* < 0.0005.

## Abbreviations

Gal-9: Galectin-9
SN: Substantia nigra
PD: Parkinson’s disease
MPTP: 1-methyl-4-phenyl-1,2,3,6-tetrahydropyridine
MPP^+^: 1-methyl-4-phenylpyridinium
SNpc: Substantia nigra pars compacta
CRD: Conserved carbohydrate-recognition domain
NDP52: Nuclear dot protein 52
TREM2: Triggering receptor expressed on myeloid cells-2
AD: Alzheimer’s disease
CSF: Cerebral spinal fluid
ICH: Intracerebral hemorrhage
Tim-3: T-cell immunoglobulin and mucin domain 3
CM: conditioned medium
GFAP: Glial fibrillary acidic protein
Iba-1: Ionized calcium-binding adaptor molecule-1
TH: Tyrosine hydroxylase
IL-1β: Interleukin-1β
IL-6: Interleukin-6
TNF-α: Tumor necrosis factor α
MIP-1α: Macrophage inflammatory protein-1α
Arg-1: Arginase-1
ROS: Reactive oxygen species
H2O2: Hydrogen peroxide
CAT: Catalase
GPx: Glutathione peroxidase
PBS: Phosphate-buffered saline
WT: Wild-type
