# Supplementary figures and images for: Galectin-9/Tim-3 pathway mediates dopaminergic neurodegeneration in MPTP-induced mouse model of Parkinson’s disease
Source: Front Mol Neurosci. 2022 Nov 21;15:1046992. doi: 10.3389/fnmol.2022.1046992 (PMC9719949; doi:10.3389/fnmol.2022.1046992)

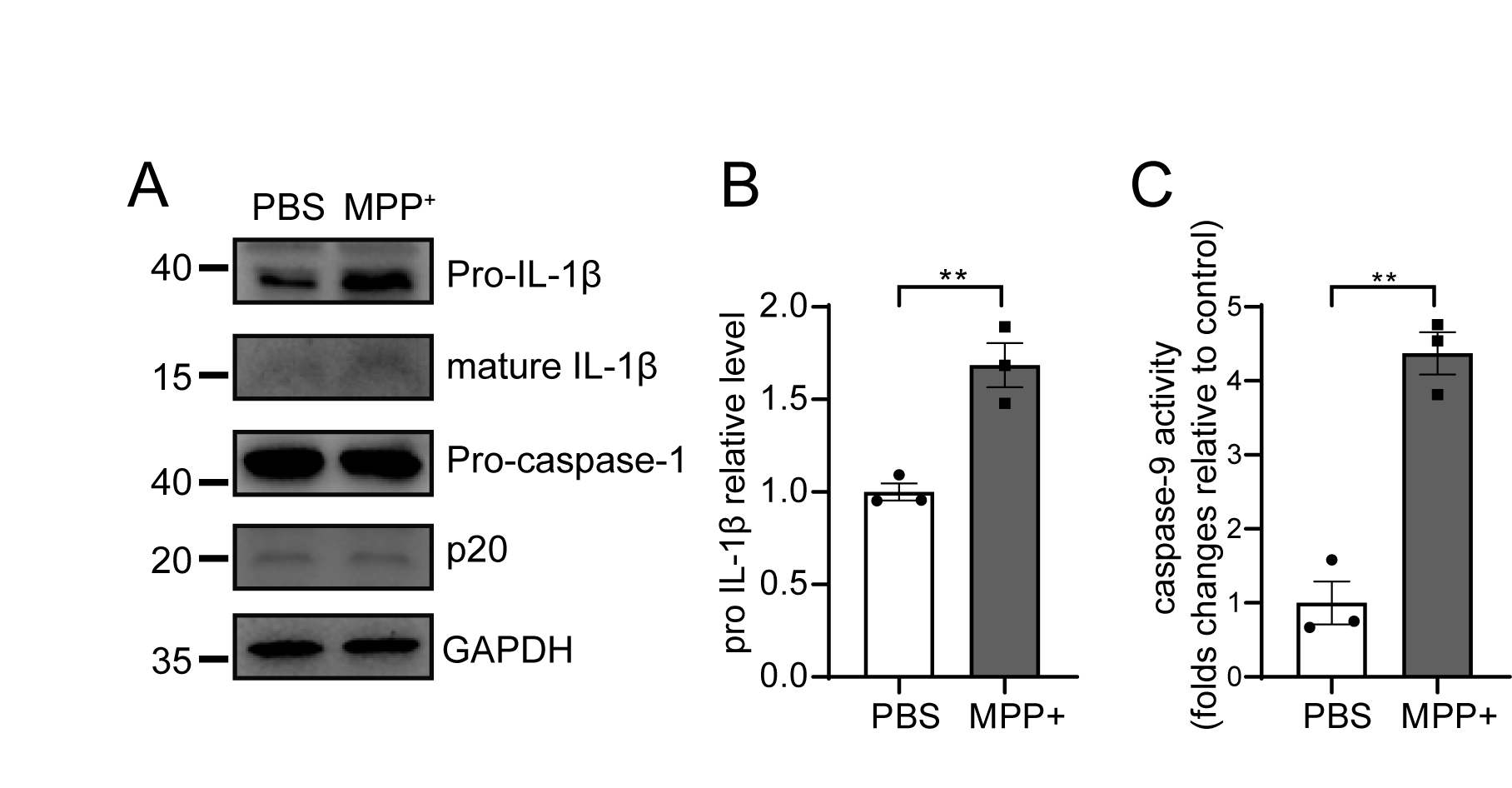

Supplement: SUPPLEMENTARY FIGURE 1 — MPP+ induces increased expression of pro-IL-1β in BV2 microglial cells. (A) Representative immunoblots of BV2 cells treated with MPP+ (500 μM, 24 h). The cellular lysates were immunoblotted with the indicated antibodies. (B) Quantification of pro-IL-1β in the cellular lysates of BV2 cells treated with MPP+. Data are presented as means ± SEM. Unpaired Student’s t-test (n = 3 independent experiments). (D) Caspase-9 activity in BV2 cells treated with MPP+ (500 μM, 24 h). Data are presented as means ± SEM. Unpaired Student’s t test (n = 3). *p < 0.05, **p < 0.005, ***p < 0.0005. [file Image_1.TIF]
